# How can tracking and tracing systems give us a look at the dark side of the tobacco market?

**DOI:** 10.1136/tc-2023-058212

**Published:** 2024-01-23

**Authors:** Filip Borkowski, Edoardo Fibbi

**Affiliations:** 1 European Commission DG CNECT, Brussels, Belgium; 2 European Commission Joint Research Centre, Ispra, Italy; 3 Department of Mathematics, KU Leuven, Leuven, Belgium

**Keywords:** surveillance and monitoring, tobacco industry, public policy, illegal tobacco products

## Abstract

**Objective:**

The aim of this work is to present possible applications of the systems of tobacco traceability for guiding local enforcement against illicit trade.

**Methods:**

The proposed three-step strategy relies on a robust regression technique and Local Moran’s I, a local indicator of spatial association, and aims at identifying retail outlets with significantly low sales compared to normal market conditions, which can indicate illegal sales activities. The ability of the method to produce alerts pointing to areas subject to illicit trade is tested on synthetic data in terms of precision and accuracy in different scenarios. Other metrics are also provided.

**Results:**

Our approach performs well under different metrics and across various levels of illicit trade prevalence, achieving a precision of 94% under the main scenario and method parametrisation.

**Conclusions:**

The proposed strategy provides high-quality leads for investigations into geographical areas disproportionately susceptible to illicit trade, potentially unveiling any form of illegal sales, including those involving products that have never entered the legal supply chain. Therefore, it can be a valuable tool for law enforcement agencies to tackle illegal sales activities. The findings of this study support also the argument in favour of expanding tobacco traceability systems downstream to the full length of the supply chain.

**WHAT IS ALREADY KNOWN ON THIS TOPIC**

- Systems of tobacco traceability are known to help in combatting the illicit trade of tobacco products. However, no previous research on potential fraud alerts is available in the public domain.

**WHAT THIS STUDY ADDS**

- Novel application of spatial econometrics and statistical analysis to the discovery of fraud in the tobacco sector.

**HOW THIS STUDY MIGHT AFFECT RESEARCH, PRACTICE OR POLICY**

- The proposed fraud discovery strategy shows that by observing legal sales at the retail level, it is possible to guide enforcement efforts successfully to the areas of illicit traders’ activity. The proposed strategy also supports the argument in favour of expanding, as much as is technically possible, the systems of tobacco traceability to the full length of the supply chain.

## Introduction

 The illicit trade in tobacco products is a global phenomenon with very significant and negative consequences on health, society and the economy. Public authorities engage considerable resources in fighting this phenomenon. Among the latest and still novel tools to limit the presence of illicit trade are tracking and tracing systems.

The launch of the first systems of this type, such as the European Union’s (EU) system of tobacco traceability, provides an unprecedented amount of new data on the supply chain of tobacco products, which can and should be explored in the quest for the best possible methods for discovering instances of illicit trade. This article suggests one of the possible avenues for analysing the available traceability data by applying spatial econometrics and statistical analysis. It proposes a novel fraud-discovery strategy aimed at signalling the areas of illicit traders’ activity to enforcement authorities. The proposed strategy targets all forms of illicit products, including those products that have never been handled within the legal supply chain.

## The context

### Illicit trade of tobacco products

The illicit trade of tobacco products is a highly lucrative criminal activity, which rests on the low production costs and the generally high taxes characterising this type of goods; the latter are imposed by governments in order to counterbalance the significant negative externalities associated with tobacco consumption. This setting offers opportunities to illicit traders that are further increased by disparities in taxation levels, reflecting, to some extent, differences in local populations’ purchasing power, which governments cannot completely disregard when setting applicable taxes.^1^

The availability of illicit tobacco products undermines the objectives of tobacco control policies. First, illicit tobacco products are less likely to comply with product regulation provisions, such as combined health warnings, bans on flavours, etc. Second, illicit tobacco products contribute to smoking initiation and facilitate tobacco consumption, in particular for young and economically vulnerable people, by providing a more affordable source of tobacco products than the legal supply chain.^2^

It is in this context that Parties to the WHO Framework Convention on Tobacco Control (FCTC) negotiated the Protocol to Eliminate Illicit Trade in Tobacco Products (the FCTC Protocol), which entered into force in September 2018. As an international treaty, the FCTC Protocol aims to eliminate all forms of illicit trade in tobacco products through a bundle of measures to be taken by countries acting in cooperation with each other.^3^

### Tracking and tracing systems

One of the key measures of the FCTC Protocol is the establishment of a global tracking and tracing regime (Article 8), consisting of national and regional systems. Parties are required to ensure that the scope of their systems is expanded to the point that all duties, taxes and other obligations have been discharged at the point of manufacture, import or release from customs or excise control.^3^

The scope of tracking and tracing systems is important for their actual use to support the fight against illicit trade. The systems may range from covering only the manufacturing part of the supply chain, along with information on shipments to the first customer who is not affiliated with the manufacturer, to expanding over the entire distribution chain, including retail outlets and the registration of sales to final consumers, or even stretching upstream to production inputs. Naturally, the broader the scope, the more possibilities are open in terms of fighting illicit trade.

A first-ever regional system of tobacco traceability, which the EU operationalised in 2019, provides a good example in this regard. The EU system requires all unit packets of tobacco products to be recorded throughout the supply chain, close to a real time, from the manufacturer to the last economic operator before the first retail outlet.^4^ This means that the EU system collects the information on shipments to individual retail outlets, but does not register retail sales to final consumers.

The 2019 World Bank review lists several potential applications of the EU system for fighting fraud which clearly rely on its scope. The following examples are provided: the discovery of duplicated unique identifiers, the discovery of the points of diversion for marked products found outside the legal supply chain, abnormal fluctuations in the manufactured or stored quantities of products, and abnormal fluctuations in quantities delivered to retail outlets.^5^ It must be noted that the cases presented in the review contradict the assertions of certain industry associations that cast doubt on whether monitoring of the manufacturing and distribution of legal and taxed products can stem the sales of illicit products. The industry argues that the tracking and tracing system can ‘only ensure a picture of the legal tobacco supply chain’ (e.g, the comments submitted by the German Smoking Tobacco Association and the Austrian Chamber of Commerce in the public feedback, which was carried out in 2017, on the EU draft secondary legislation laying down detailed rules of the EU system of tobacco traceability; see 6). Our work provides an argument against this claim.

## Methodological framework

The main goal of this article is to show how a picture of the legal tobacco supply chain can actually be informative about illicit trade, the ‘dark’ side of the tobacco market, even if illicit products have not been diverted from or inserted into the legal supply chain at any point. The following analysis therefore focuses on quantities observable at the retail level.

As explained above, the EU system provides for a high-spatial granularity of traceability data, that is, products are traced up to individual retail outlets, based on dispatch messages reportable by distributors.

This article proposes a novel fraud-discovery strategy aimed at signalling to enforcement authorities the areas of illicit traders’ activity. The proposed strategy rests on a couple of common-sense but methodologically important assumptions. Although illicit trade is a widespread phenomenon, it can be expected to be heterogeneous in time and with some areas either more or less exposed on a temporary basis to illicit traders, whose individual distribution networks are expected to be interrupted occasionally by enforcement authorities. At the same time, the legal distribution chain is expected to be largely reliable and always ready to supply the quantities needed by consumers, who are expected to exhibit stable demand, which reflects the addictive character of tobacco products. The demand may, however, be subject to certain annual seasonality as well as to a long-term downward trend reflecting the growing awareness of the harmfulness of tobacco products. The demand is satisfied by both licit and illicit sources. These assumptions are basic, but important for the logic of the proposed strategy, which is preconditioned by the existence of a tracking and tracing system collecting the information on individual retail outlets.

The proposed strategy is implementable in three steps. In the first step, historical data for all retail outlets active in a given area are used to generate predictions of sales (quantities of shipped products) for each retail outlet. Any modelling process can be expected to draw on a series of explanatory and control variables to achieve the maximum accuracy of its predictions, which should nonetheless deliberately miss the effect of illicit trade. Among possible controls, the most obvious ones are a time trend, seasonality, a measure of local competition, shipment ruptures or retail closures. Monthly seasonality may help to capture changes in sales that are due to factors affecting consumption such as weather, annual tax changes or New Year’s resolutions.^7^ Local competition is an element reflecting how many retail outlets compete in a given (sub)area. The presence of shipment ruptures may also necessitate its own control variable. Such a control will at least partially address the problem of data originating from tracking and tracing systems in which a product’s history ends with its dispatch to a retail outlet, as is the case in the EU. The usefulness of this control will depend on the time frame adopted for collecting individual observations, which in turn will depend on an average frequency of shipments to retail outlets.

In the second step, the sales volumes (quantity of shipped products) predicted in the first step should be set against the true observed values. The resulting deltas should be noted down as follows:


(1) $$\Delta_{it}\;=\;x_{it}\;-{\hat{x}}_{it}$$

where $x_{it}$ is the observed volume for the *i*th outlet at time *t*.

The last step consists of calculating a local measure of spatial autocorrelation. For this, it is proposed to rely on Local Moran’s I, a well-known indicator belonging to the class of local indicators of spatial association (LISAs), which allow for the investigation of pockets of spatial non-stationarity and local outliers.^8^ We recall that the Local Moran’s I is defined by:


(2) $$I_{i}=\frac{z_{i}\sum_{j}w_{ij}z_{j}}{\sum_{j}z_{j}^{2}/\left(n-1\right)}$$

where $w_{ij}$ is the weight for the pair of units (i,j) and $z_{i}$ denotes the residual of the variable of interest for unit i, which in our case is $\Delta_{it}$, for a fixed time *t*, as defined in (1). It is assumed that, locally, illicit trade is unpredictable but discoverable. For each given location, LISA values allow for the computation of that location’s similarity to its neighbours and for testing its significance.^9^ Intuitively, $I_{i}$ is a weighted measure of association between the deltas of retailer i and those of its neighbours, with the neighbouring relation determined by the weights defined in the next section. A positive $I_{i}$ indicates that retailer i behaves similarly to its neighbours in terms of $\Delta_{it}$, whereas a negative value means that $\Delta_{it}$ stands out locally. In the context of fraud discovery, the so-called ‘cold spots’ are of particular interest. These are locations with large negative values for the deltas, surrounded by similarly low values of the deltas in neighbouring locations, leading to a low–low pattern and thus positive $I_{i}$ . Finding such cold spots should guide enforcement authorities towards areas where the actual volumes are significantly below the expected values. The low–low pattern indicates that the unexpectedly low sales are present in the entire neighbourhood. This also indicates the existence of a common factor, with the hypothesis that this factor is an inflow of illicit products into the area. At the same time, issues affecting an individual outlet are unlikely to cause a low–low pattern. These would be expected to result in the low–high pattern, in which the neighbouring outlets compensate for a drop in the sales of an individual outlet. Cold spots are detected for positive values of Local Moran’s I that are closer to +1 (within the test’s range of −1 to +1) for retail outlets, with the negative deltas calculated in the second step.

## Simulation study

### Data generation

For reasons of confidentiality, it has not been possible to use any authentic traceability dataset in preparing this article. Therefore, a synthetic dataset was generated. Its spatial dimension was constructed as a hexagonal tiling, consisting of 29 columns and 29 rows, for a total of 826 fields. As for the temporal dimension, 122 biweekly delivery periods were simulated, spanning 5 years and 1 month.

On the demand side, each field is initialised with 1000 units, subject to a steady contraction of approximately 2% per year. The assumed contraction rate approximates the actual decrease by 40% of licit cigarette consumption observed in the EU27 Member States between 2005 and 2021.^10^ The demand also exhibits a seasonality of +4% from May to August and −4% from November to February, in line with the winter–summer seasonality pattern established in the literature.^7^ The demand’s measured volumes include independent random errors following a centred normal distribution with SD of 1%.

The supply side consists of 196 legal retail outlets evenly distributed over the hexagonal map and a fixed number of illicit traders, which are randomly allocated to the fields unoccupied by the legal ones. The exact number of illicit traders is defined as a fraction of the whole market. We ran simulations for a 5%, 10% and 15% fraction, corresponding to 10, 22 and 35 illicit traders, respectively. Each illicit trader faces a 4% probability of forced reallocation in each delivery period. Both legal retail outlets and illicit traders serve the entire market, but their market clout decays exponentially with distance and is equal to *e*^−*d*^, where *d* is the hex-distance, that is, the minimum number of adjacent hexagons that have to be traversed to get from a cell to another, including the endpoint. The market clout allows for the establishment of the market shares of each retail outlet and illicit trader in the demand generated by each field.

**Figure 1:**
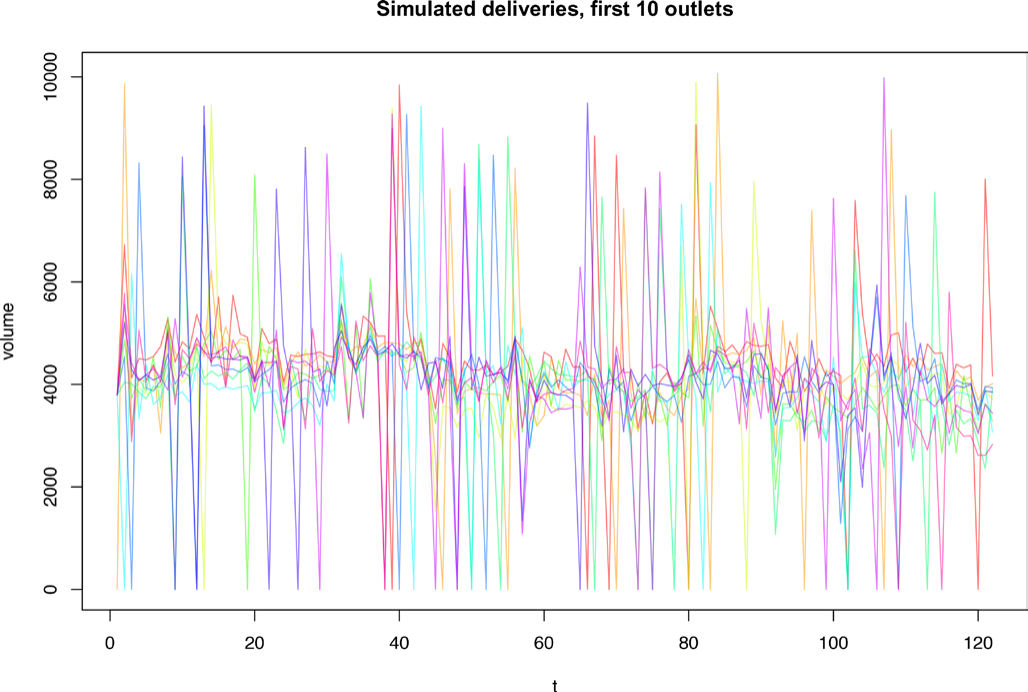
Volumes of deliveries for first 10 retail outlets, each denoted by a different colour.

Supplies’ volumes emulate the tracking and tracing systems, in which a product’s history ends with its dispatch to a retail outlet. They are influenced by the stockpiling of products by the retail outlets, which at any given period aim to keep enough products to satisfy the demand in the current and the next one and a half periods. In each period, the retail outlets, independently of each other, face a 4% probability of delivery interruption. However, such a delivery interruption never repeats itself over two consecutive periods. The retail outlets make up for missed deliveries with increased orders for the subsequent period. Furthermore, individual outlets may be temporarily closed for random reasons. These closures are short-lived and have a 12% probability of occurring in each period, with a 4% and an 8% probability of the closure for 10% and 5% of the time in a given period, respectively. Figure 1 shows the simulated deliveries for 10 retailers.

### Application of the fraud-discovery strategy

Predictions of sales (quantities of shipped products) for each retail outlet were generated by means of a pooled linear regression of the following model:


(3) $$\begin{array}{ll} x_{it}= & \beta_{1}+\beta_{2}t+\beta_{3}md_{it}+\beta_{4}ed_{it}+\\ \beta_{5}comp_{it}+\sum_{k=1}^{11}\beta_{5+k}m_{k}+\varepsilon_{it} \end{array}$$

with the indices i = 1, 2,…, 196 and t = 3, 4,…, 122 identifying the retail outlet and time period, respectively.

In the above model, the volumes of shipped products per retail outlet ($x_{it}$) are regressed against a set of control variables: time period (*t*), level of competition (*comp*), dummy variables representing missed (*md*) and extra catch-up (*ed*) deliveries, and 11 dummy variables representing the first 11 months of the year (*m*_1_*–m*_11_), to account for seasonality. The first two periods were skipped in the regression to enable the full scope of fluctuations in shipments, which are generated by the stock policy adopted by the retail outlets, to set in. The level of competition was approximated with the number of retail outlets located within the distance of three hexes. The variables representing missed and extra catch-up deliveries were observed from the dataset in situations where no delivery was shortly followed by a delivery re-establishing the desired stock levels.

We estimated model (3) through a robust regression procedure, fitting an MM-type estimator,^11 12^ which ensures high resistance to outliers while still being highly efficient at the uncontaminated linear model with normal independent and identically distributed errors.

In the case of real-world data, it would be, of course, advisable to look into potential specification errors as well as sources of autocorrelation that might be unrelated to the presence of illicit traders, including demographic characteristics. For example, assuming that those sources are likely to be retail outlet specific (eg, petrol station vs supermarket vs tobacco retailer), the addition of time-invariant fixed effects could improve the precision of a regression model.^13^

The predictions obtained were compared with the observed volumes per retail outlet. The resulting deltas were used to calculate the values of Local Moran’s I, where each retailer was analysed within its neighbourhood of three hexes. In more detail, in equation (2), we put $w_{ij}=1/n_{i}$, $n_{i}$ being the number of i’s neighbours, if j is within three cells from i, and $w_{ij}=0$ otherwise. The retail outlets were marked as suspected to be suffering from the presence of illicit traders in their vicinity whenever the value of Local Moran’s I was found to be positive at a 1% significance level for two consecutive periods. This type of parametrisation is suggested when enforcement authorities have limited resources and thus prefer to receive highly probable alerts at the cost of accepting a higher rate of type II errors.

### Assessment method

Given the costs of investigations, our methodology aims at finding high-reliability geographical areas with patterns of illicit trade, rather than isolated frauds. In our model, interactions have infinite (although exponentially decaying) range, but in practice, it is reasonable to suppose that anti-fraud authorities set a finite search radius around alerts. Therefore, we investigated how the methodology behaves with respect to the minimal number of illicit traders *n* within a given search radius *r* (in hex-distance) from a legal outlet that has triggered an alert. Given two integers n*>*0 and r*>*0, for all time periods *t,* we computed the number of true positives (TP), false positives (FP), true negatives (TN) and false negatives (FN) according to these definitions:

- TP: alerted retailers having at least *n* illicit traders within a radius *r* from them.
- FP: alerted retailers having less than *n* illicit traders within a radius *r* from them.
- TN: unalerted retailers having less than *n* illicit traders within a radius *r* from them.
- FN: unalerted retailers having at least *n* illicit traders within a radius *r* from them.

The counts of TP, FP, TN and FN were obtained for different combinations of (n,r) – r = 1,…, 4 and n= 1,…, 3. From these, common metrics (e.g, precision, recall, accuracy) were computed to assess the method. Their definitions are in the online supplemental appendix.

### Results

**Figure 2:**
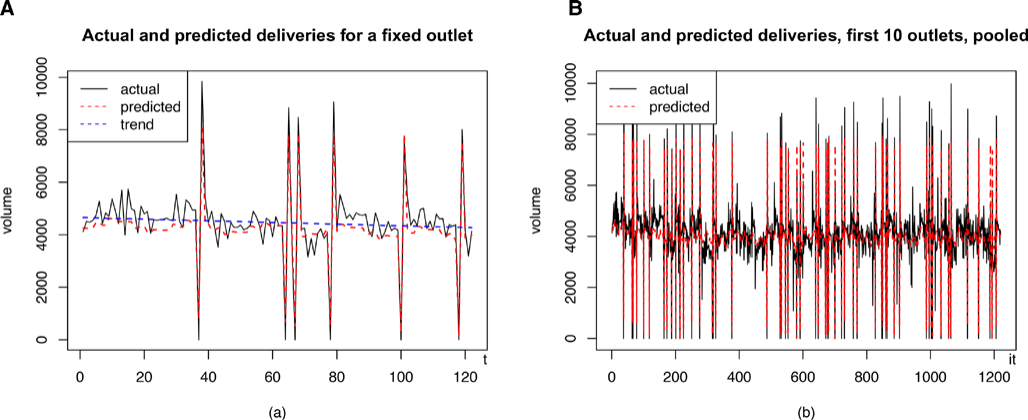
Comparison between actual and predicted volumes, represented with a black continuous line and a red dashed line, respectively. The model reproduces well-important features of the data, such as the effect of missed and extra deliveries (peaks and troughs in the volumes), seasonality, etc, without overfitting. (A) Fixed retail outlet and (B) pooled data.

In the scenario with a 10% proportion of illicit traders, the pooled regression produced an adjusted R^2^ of 0.84, which is consistent with the number of illicit traders and the amount of noise added in the data-generating process. The Durbin-Watson statistic is low (d=1.86), confirming the presence of positive spatial correlation in the synthetic dataset. In the scenarios with 5% and 15% prevalence, the regression produced, respectively, $R^{2}=0.88$, d=2.07 and $R^{2}=0.79$, d=1.68. Figure 2 compares predictions with actual data.

**Figure 3:**
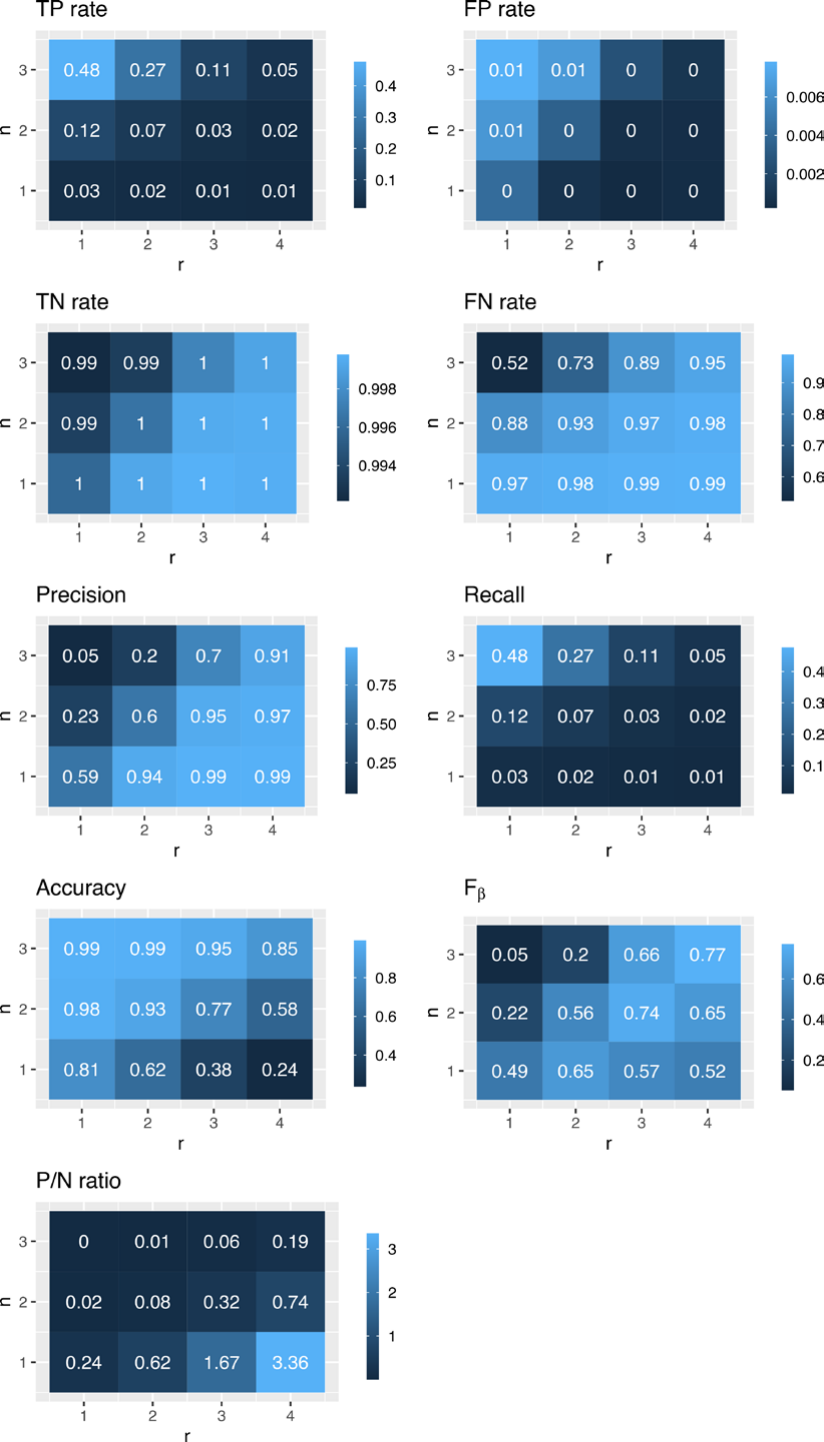
Heatmap representations of various statistics, for different combinations of search radius, r, and minimum number of illicit traders within the search radius, n. Base scenario (market share of illicit trade: 10%). Darker colours correspond to lower values. FN, false negative; FP, false positive; P/N ratio, ratio between positives and negatives; TN, true negative; TP, true positive.

Figure 3 shows the results obtained for the 10% scenario, while the other two scenarios can be found in the online supplemental material. Generally, the strategy works well (in terms of precision and accuracy) when the search radius *r* and the number of illicit traders *n* around an alert are large enough, as expected. Precision and accuracy are high for a good number of choices of (n,r), while recall is usually low.

Although a considerable number of illicit traders remains uncovered, the alerts produced by our strategy are very likely to point to areas with illicit trade. This is a desirable feature in an anti-fraud context, since investigations come at a cost: having a few high-quality leads is better than having more of them but also many false alarms, or having no guidance at all.

Table 1 and the plots in the online supplemental appendix show that our approach is sufficiently stable with respect to different levels of illicit trade.

**Table 1:** Comparison of the three scenarios, with different proportions of illicit trade

**Illicit traders’ (i.t.) market share**	**5%**	**10%**	**15%**
Number of i.t.	10	22	35
Total number of alerts over 120 periods	180	194	271
Total number of i.t. under alerts (r=1)	106	139	185
Total number of i.t. under alerts (r=2)	144	226	367
Precision over 120 periods (n=1 and r=2)	92.22%	94.32%	99.63%
Accuracy over 120 periods (n=1 and r=2)	80.80%	62.28%	46.49%

The proposed methodology behaves well in terms of precision in all three cases. Accuracy is acceptable and degrades only at higher levels of illicit trade.

**Figure 4:**
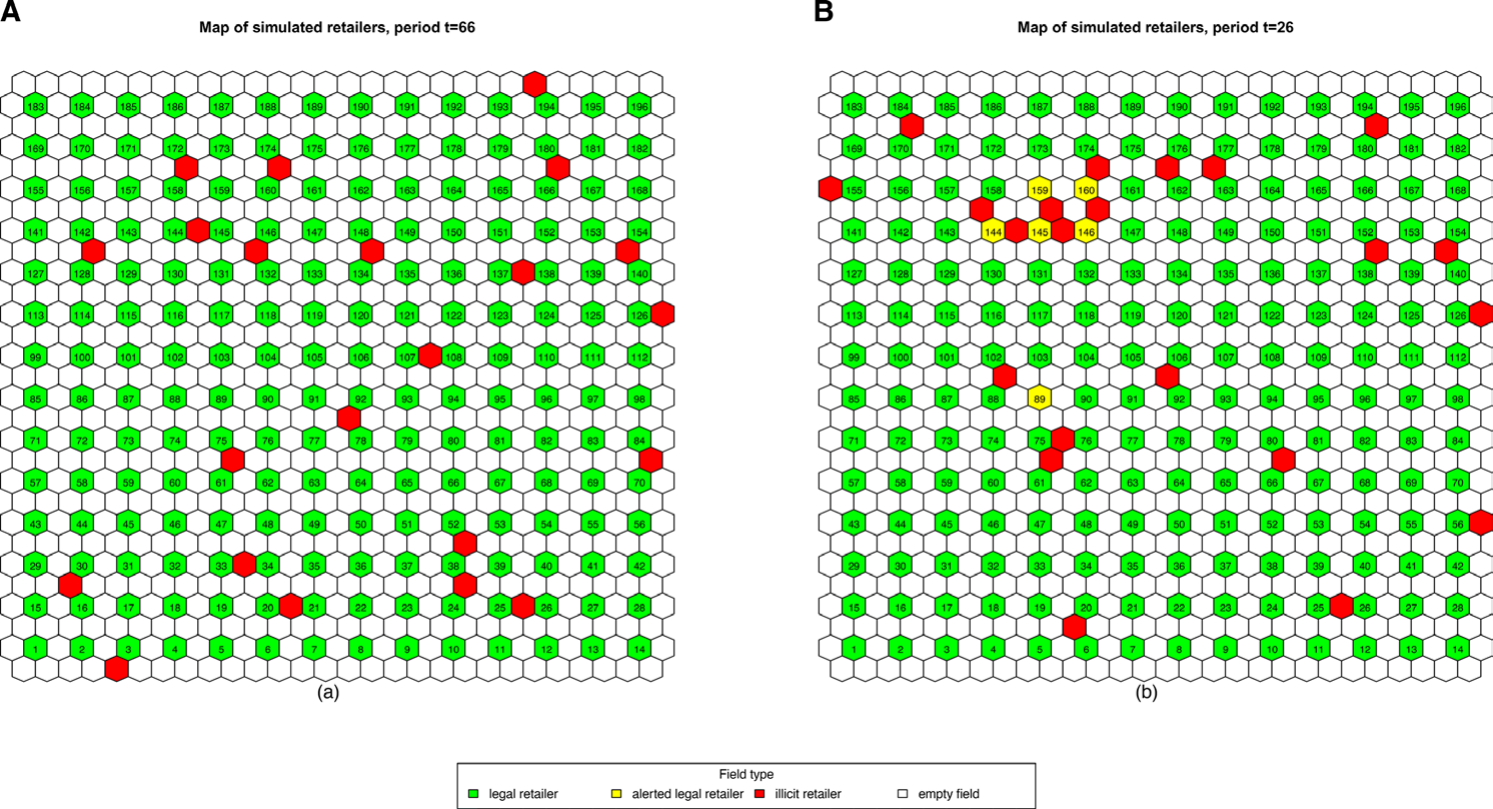
Graphical display of simulated outlets and illicit retailers on the hexagonal grid. In green, legal retail outlets; in yellow, legal retail outlets that triggered an alert; in red, illicit traders. Legal retailers occupy numbered fields, while white tiles represent unoccupied locations. We can see that areas with a higher concentration of illicit trade are correctly identified. (A) Well-spread illicit trade and (B) high-density areas of illicit trade.

Overall, from our analysis, it seems that the methodology is coherent with the anti-fraud needs and objectives. This is confirmed also by a visual inspection of the results, showing how the proposed strategy leads to the correct identification of those retail outlets that are particularly exposed to the activity of illicit traders in their vicinity. Not surprisingly, a higher local concentration of illicit traders yields better performances (figure 4B). Period t=66 is a good example of when no discovery alerts are produced due to the fact that illicit traders are relatively well spread over the entire territory (figure 4A).

The above analysis of quantities of shipped products can be extended in multiple ways, in particular by using real data, which, in the EU system, include the full decomposition of all the shipped quantities by product type, brand, presentation, etc. This means that a fraud alert can be interpreted more fully. For example, if the fluctuations triggering an alert concern a premium brand, it is likely that illicit traders distribute a counterfeit or genuine product of that brand. If cheaper products are disproportionately affected, it may indicate the inflow of the so-called illicit whites.

## Conclusions

Our work shows that, by observing legal sales at the retail level, it is possible to guide successfully enforcement efforts to the areas of illicit traders’ activity. Importantly, the proposed strategy allows for the discovery of any form of illicit trade, including those illicit products that have never been handled within the legal supply chain. The discovery is possible for both historical and current data, where the latter is obviously the most interesting for enforcement purposes.

The proposed strategy provides high-quality leads for investigations into those geographical areas disproportionately susceptible to illicit trade. However, our approach relies on two assumptions, notably the relative instability over time and the uneven distribution of illicit trade at the local level. Therefore, it may be successful in fighting illicit traders only where the latter are already under sufficient enforcement pressure to destabilise their local distribution networks temporarily.

Our results support the argument in favour of expanding, as much as is technically possible, the systems of tobacco traceability to the full length of the supply chain. The tracking and tracing system adopted in the EU represents a minimum workable solution, insofar as it includes information on shipments by distributors to retail outlets. However, it must be noted that the system’s expansion to the registration of the final sales to consumers could lead to a multifold increase in the granularity and hence the precision of the data, while necessary time series could be collected in much shorter times.

Finally, in technical terms, the proposed strategy, once implemented, should not represent any major computation obstacles as the data dimensions are limited to the number of retail outlets and time periods in a dataset. Also, given that track-and-trace systems are legislated to limit the illicit trade (eg, see 14), there should be no legal obstacles for enforcement authorities to use these systems in line with their objective.

## Supplementary material

10.1136/tc-2023-058212 online supplemental file 1

## Data Availability

Data are available upon reasonable request.
