# Supplementary material for: How can tracking and tracing systems give us a look at the dark side of the tobacco market?
Source: Tob Control. 2024 Jan 23;34(3):e058212. doi: 10.1136/tc-2023-058212 (PMC12128766; doi:10.1136/tc-2023-058212)
Supplement: online supplemental file 1 [file tc-34-3-s001.pdf]

Supplementary material to the article "How can tracking and tracing systems give us a look at the dark side of the tobacco market?"

Filip Borkowski[1], and Edoardo Fibbi[2, 3, *]

[1]European Commission, DG SANTE, Rue Breydel 4, 1040 Bruxelles, Belgium
[2]European Commission, Joint Research Centre, Via Enrico Fermi 1479, 21027 Ispra, Italy
[3]KU Leuven, Department of Mathematics, Celestijnenlaan 200b, 3001 Leuven, Belgium

[*]Corresponding author. E-mail: edoardo.fibbi@ec.europa.eu; postal address: office 26B 02/244, via Enrico Fermi 1479, 21027 Ispra, Italy

SUPPLEMENTARY MATERIAL

A.1 Model diagram

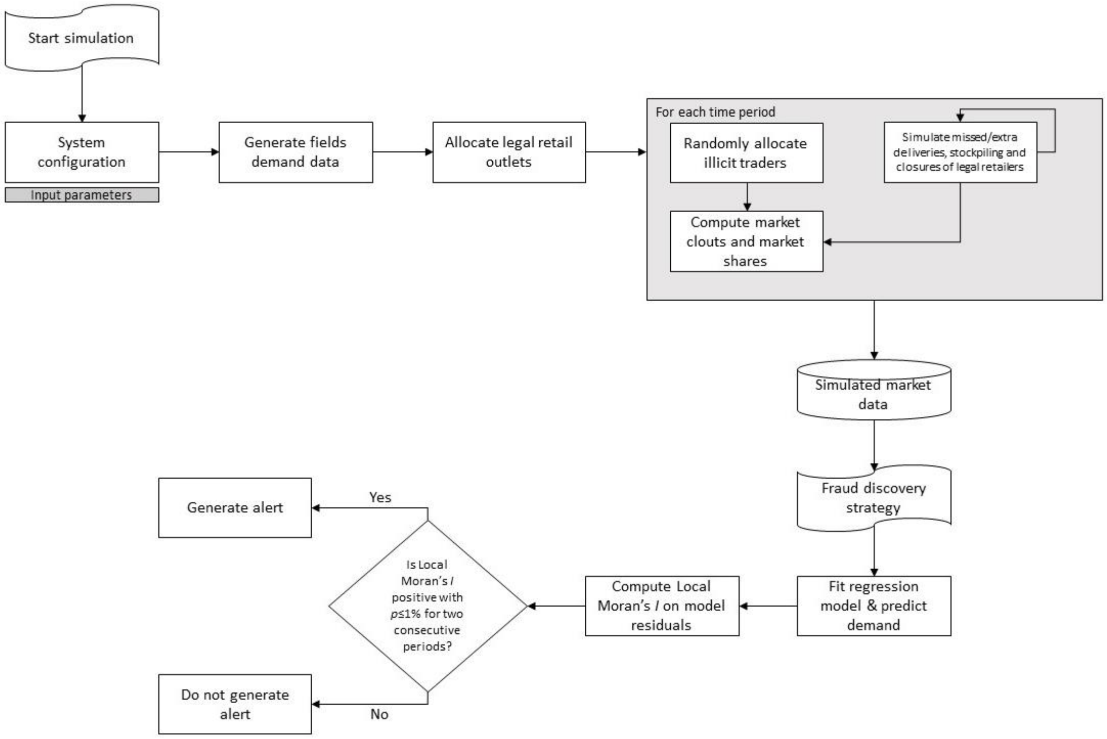

A.2 Assessment metrics

We recall that a confusion matrix consists in a two-by-two table containing true positive (TP), false positive (FP), true negative (TN) and false negative (FN) counts, usually having the actual positive / negative condition as rows and the predicted condition as columns. In other terms, it is a contingency table obtained from the actual versus predicted condition. From the confusion matrices obtained for each pair (n, r), some common metrics are computed (precision, accuracy, F_β-scores, as well as TP, FP, TN and FN rates), whose definitions follow:

- Precision: $TP/(TP + FP)$, higher is better.
- Accuracy: $(TP + TN)/(P + N)$, higher is better.
- F_β-score: $(1 + \beta^2)\ precision \cdot recall/(\beta^2\ precision + recall)$, higher is better. In our simulations $\beta = 0.1$.
- TP rate (sensitivity or recall): $TP/(TP + FN)$ higher is better.
- TN rate (specificity): $TN/(TN + FP)$, higher is better.
- FP rate (false alarm rate): $FP/(TN + FP)$, lower is better.
- FN rate (miss rate): $FN/(TP + FN)$, lower is better.

A.3 Simulation results with different levels of illicit trade

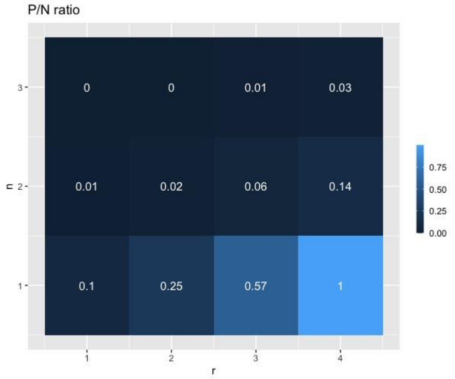

(a) Class balance.

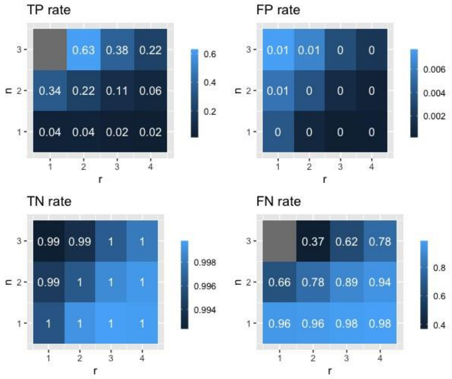

(b) TP, FP, TN and FN rates.

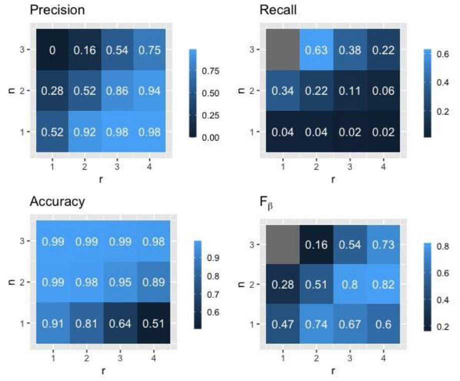

(c) Metrics. F_β computed for $\beta = 0.1$.

Figure 5: Heatmap representations of various statistics, for different combinations of search radius, r, and minimum number of illicit traders within the search radius, n. Low-diffusion scenario (market share of illicit trade: 5%).
Grey areas in Figures 5b and 5c correspond to the case in which there were no positives (according to the definition given in Subsection 4.3) in the simulations.

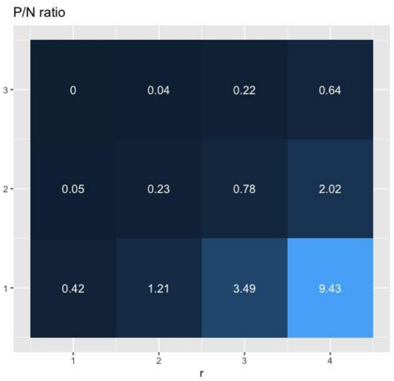

(a) Class balance.

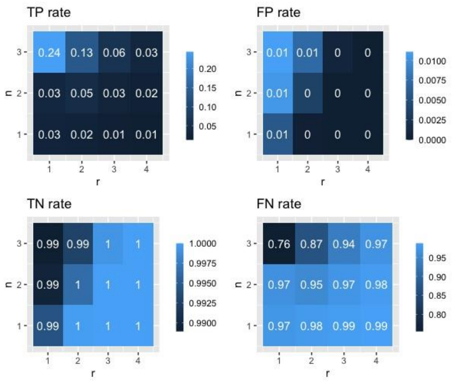

(b) TP, FP, TN and FN rates.

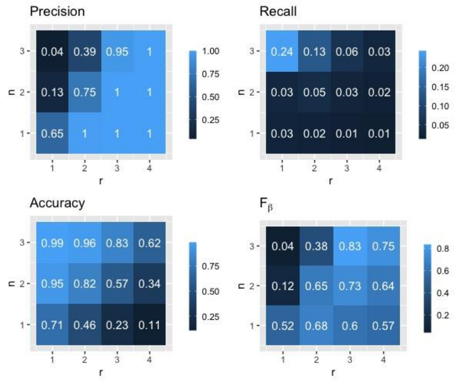

(c) Metrics. F_β computed for $\beta = 0.1$.

Figure 6: Heatmap representations of various statistics, for different combinations of search radius, r, and minimum number of illicit traders within the search radius, n. High-diffusion scenario (market share of illicit trade: 15%).

A.4 Technical specifications

- The code was developed in R 4.2.1 and run on a Red Hat Enterprise Linux (RHEL) 8.6 machine.

- Hardware specifications: Intel® Xeon® Gold 6230 @2.10 GHz CPU, 64 GB RAM.
- Runtime: data simulation in the 10% scenario required about 13.8 h, while the discovery strategy required about 1 min 50 s.
- R packages used: `schoolmath` (0.4.1), `sf` (1.0-9), `spdep` (1.2-7), `MASS` (7.3-57), `robustbase` (0.95-0), `ggplot2` (3.3.6), `gridExtra` (2.3), `latex2exp` (0.9.5).
- Random numbers were generated using the default random number generator of R, based on the Mersenne Twister algorithm.
- The original code may be provided upon request.
